# Bison movements change with weather: Implications for their continued conservation in the Anthropocene

**DOI:** 10.1002/ece3.9586

**Published:** 2022-12-08

**Authors:** Nicholas A. McMillan, Samuel D. Fuhlendorf, Barney Luttbeg, Laura E. Goodman, Craig A. Davis, Brady W. Allred, Robert G. Hamilton

**Affiliations:** ^1^ Department of Agronomy and Horticulture School of Natural Resources University of Nebraska‐Lincoln Lincoln Nebraska USA; ^2^ Natural Resource Ecology and Management Oklahoma State University Stillwater Oklahoma USA; ^3^ Integrative Biology Oklahoma State University Stillwater Oklahoma USA; ^4^ W.A. Franke College of Forestry & Conservation University of Montana Missoula Montana USA; ^5^ Joseph H. Williams Tallgrass Prairie Preserve Pawhuska Oklahoma USA

**Keywords:** climate change, conservation, grasslands, landscape, movement ecology, ungulates, weather variability

## Abstract

Animal movement patterns are affected by complex interactions between biotic and abiotic landscape conditions, and these patterns are being altered by weather variability associated with a changing climate. Some animals, like the American plains bison (*Bison bison* L.; hereafter, plains bison), are considered keystone species, thus their response to weather variability may alter ecosystem structure and biodiversity patterns. Many movement studies of plains bison and other ungulates have focused on point‐pattern analyses (e.g., resource‐selection) that have provided information about where these animals move, but information about when or why these animals move is limited. For example, information surrounding the influence of weather on plains bison movement in response to weather is limited but has important implications for their conservation in a changing climate. To explore how movement distance is affected by weather patterns and drought, we utilized 12‐min GPS data from two of the largest plains bison herds in North America to model their response to weather and drought parameters using generalized additive mixed models. Distance moved was best predicted by air temperature, wind speed, and rainfall. However, air temperature best explained the variation in distance moved compared to any other single parameter we measured, predicting a 48% decrease in movement rates above 28°C. Moreover, severe drought (as indicated by 25‐cm depth soil moisture) better predicted movement distance than moderate drought. The strong influence of weather and drought on plains bison movements observed in our study suggest that shifting climate and weather will likely affect plains bison movement patterns, further complicating conservation efforts for this wide‐ranging keystone species. Moreover, changes in plains bison movement patterns may have cascading effects for grassland ecosystem structure, function, and biodiversity. Plains bison and grassland conservation efforts need to be proactive and adaptive when considering the implications of a changing climate on bison movement patterns.

## Graphical abstract

Animal movement patterns are affected by complex interactions between biotic and abiotic landscape conditions, and these patterns are being altered by weather variability associated with a changing climate. Some animals, like the American plains bison (*Bison bison* L.; hereafter, plains bison), are considered keystone species, thus their response to weather variability may alter ecosystem structure and biodiversity patterns. Here, we show that bison movement distances were best predicted by air temperature, wind speed, and rainfall. The strong influence of weather and drought on plains bison movements observed in our study suggest that shifting climate and weather patterns will likely affect plains bison movement patterns, further complicating conservation efforts for this wide‐ranging keystone species.

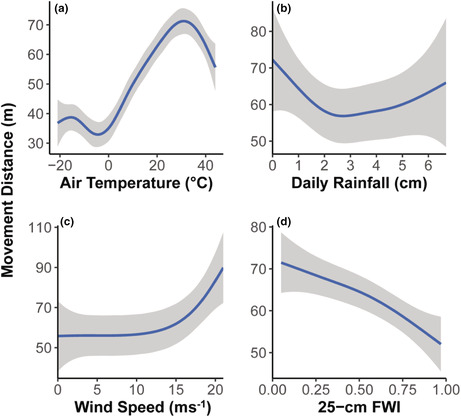

## 1 INTRODUCTION

The movement patterns of many large ungulates are entwined with the cyclical rhythms of their environment (e.g., seasonal patterns of vegetation phenology), which are in part driven by climate (i.e., long‐term weather trends) and weather (McMillan et al., 2021; Owen‐Smith & Goodall, 2014; Schmidt et al., 2016). Anthropogenic climate change is altering global weather patterns (Cai et al., 2014) and complicating conservation and management efforts for many species (Stenseth et al., 2002; Thomas, 2010), including ungulates. Ungulate movement patterns, in particular, can be strongly influenced by weather patterns (Augustine, 2010), often moving long‐distances to follow weather‐induced changes in forage quality (Fryxell & Sinclair, 1988; Holdo et al., 2009; Mueller et al., 2008). Due to anthropogenic pressures (e.g., social conflict or land conversion), some large ungulates like American plains bison (*Bison bison* L.), elk (*Cervus elaphus* L.), and elephants in Africa (*Loxodonta africana* Blumenbach) and Asia (*Elephas maximus* L.) have mostly become restricted to roaming small, highly regulated conservation areas relative to the landscapes they once inhabited (Frair et al., 2005; Geremia et al., 2019; Shaffer et al., 2019). Many large ungulates are also predicted to experience climate change‐induced range boundary shifts (Thomas, 2010), which further complicates ongoing conservation efforts for those species already restricted to small areas due to human encroachment. While many studies have addressed how ungulate habitat selection changes in response to physical landscape features (e.g., phenology, topography, roads; Geremia et al., 2019; Merkle et al., 2016; O'Shaughnessy et al., 2014), relatively few have attempted to address how their movement—regardless of the underlying landscape—is affected by weather (Boyers et al., 2019; Schmidt et al., 2016; Sheppard et al., 2021). Understanding how ungulates respond to weather is critical for predicting the long‐term consequences of climate change and informing their conservation in the Anthropocene.

The American plains bison (*Bison bison* L.; hereafter plains bison) is a large ungulate grazer that historically roamed nearly all of North America, spanning the entire Great Plains and portions of the eastern and western regions of the continent (Hall, 1981). Plains bison were and continue to be integral to the culture of the Indigenous Peoples of the Great Plains, yet written records documenting their historical movement patterns are sparse (Hornaday, 1889; Shaw, 1995). Since their near extinction in the late 19th and early 20th century, most plains bison have been relegated to small, restricted, and structurally homogenous landscapes that are quite different from the expansive, heterogeneous landscapes they once roamed (Fuhlendorf et al., 2018; Sanderson et al., 2008). Some have even suggested that the plains bison may be ecologically extinct due to differences between their historical landscape and those they currently inhabit (Freese et al., 2007; Fuhlendorf et al., 2018; Soulé et al., 2003). Therefore, very few studies of plains bison have addressed bison behavior and movement across large landscapes managed with historically relevant processes (e.g., promoting structural heterogeneity with fire) that are critical to broader biodiversity conservation goals.

Growing social conflict surrounding plains bison movement (i.e., movement out of conservation areas onto private lands) has created tension between agricultural and conservation groups that are likely going to get worse with changing climate (Plumb et al., 2009). For example, each year several thousand plains bison at Yellowstone National Park (899,116 ha) annually disperse from the park, inciting conflict between the U.S. National Park Service and surrounding landowners (Plumb et al., 2009). Some plains bison restoration projects across the United States have also been subject to similar social conflicts, largely driven by unauthorized movement—or fear thereof—onto neighboring private lands (Davenport, 2018). Climate change is increasing the intensity and frequency of extreme weather and drought events, and is likely contributing to these socioecological conflicts (Fuhlendorf et al., 2018). Plains bison restoration projects across North America are also broadly justified based on hypothesized keystone species effects (McMillan et al., 2019). However, the current and historical ecological effects of many herbivores—including bison—are strongly tied to environmental factors including thermal conditions and climate change (Fuhlendorf et al., 2018). However, with the exception of habitat and forage preferences (Allred et al., 2013; Craine et al., 2015), how plains bison movements are affected by weather extremes, including drought, is entirely unknown. Therefore, understanding how plains bison respond to external environmental stressors, like weather and drought, would directly inform critical—and difficult—conservation or restoration decisions ongoing across North America in‐light of continued climate change.

Weather and drought are hypothesized to drive plains bison movement directly through physiological stress (Allred et al., 2013), as well as indirectly by altering the quality and quantity of resources needed for survival and maintenance (Owen‐Smith & Goodall, 2014). Plains bison have also shown seasonal movement patterns that may be driven, in‐part, by weather (McMillan et al., 2021). However, much of the plains bison movement literature is limited to point‐pattern investigation (e.g., resource‐selection) focused on how habitat configuration and composition affects movement across a landscape. While the aforementioned studies can be useful in determining habitat use, we know of only one study that has attempted to address how plains bison move through space irrespective of where they are on the physical landscape (McMillan et al., 2021). Moreover, although weather may influence the energetic costs of movement (e.g., increased wind speed being linked to an increased energetic cost of movement; Halsey, 2016), few studies have confirmed or described the effect of weather on movement for other large mammals of conservation concern (Schmidt et al., 2016; Sheppard et al., 2021; van Beest et al., 2012). Air temperature and plant‐available soil moisture (a drought indicator), in particular, can both strongly influence forage distribution, quantity, and quality. Severe drought is characterized by low soil moisture that extends deep within the soil profile (Basara et al., 1998), and likely has a significant influence on plains bison movement. As ungulate grazers, forages can also provide plains bison with most of their daily water requirement (Kay, 1997; King, 1983). Forage moisture content is tied to soil moisture, and during severe drought, ungulate grazers largely depend on permanent or ephemeral water sources to meet their physiological needs (Kay, 1997). Historical accounts of movement patterns in plains bison suggest they may have traveled long distances, and for multiple days without water (Hornaday, 1889). Although previous studies have attempted to address the influence of water distribution on plains bison resource selection (Kohl et al., 2013), no studies have specified how sensitive their movements might be to drought. Given increasing social conflict, landscape fragmentation, and climate change; how these large and important herbivores respond to weather and drought may determine the feasibility of maintaining bison herds throughout the Great Plains during the Anthropocene.

We analyzed a dataset from two of the largest plains bison herds in North America, the Wichita Mountains Wildlife Refuge and the Tallgrass Prairie Preserve bison herds, to investigate how plains bison movement is affected by weather. We specifically set out to determine how weather (i.e., wind speed, wind direction, relative humidity, rainfall, air temperature, solar radiation) as well as drought affects plains bison movement distance (i.e., a primary path‐signal). We hypothesized that plains bison movements would closely track air temperature more than other weather parameters because of its effect on resource selection (Allred et al., 2013) and forage quality (Owen‐Smith & Goodall, 2014; Pilarski, 1999; Sage & Kubien, 2007). We also hypothesized that plains bison movement would be more affected by severe than moderate drought conditions given that recent studies (Kohl et al., 2013) and historical accounts (Hornaday, 1889) suggest they may not be sensitive (behaviorally) to surface water abundance.

## 2 METHODS

### 2.1 Study sites

Data for this study were collected across two sites in Oklahoma, USA that vary considerably in their topography, vegetation, and climate: The Nature Conservancy's Joseph H. Williams Tallgrass Prairie Preserve (hereafter, Tallgrass Prairie Preserve) and the United States Fish and Wildlife Service's Wichita Mountains Wildlife Refuge (Table 1). The Tallgrass Prairie Preserve is divided into two distinct units based on the dominant grazer (cattle or bison), and our study focused on data collected in the 9400‐ha bison unit where approximately 2500 bison are allowed to freely graze year long. Most of the Tallgrass Prairie Preserve is managed with fire under the patch‐burning management paradigm that is focused on restoring structural heterogeneity on the landscape (Hamilton, 2007). Fire is applied at various times throughout the year in the Tallgrass Prairie Preserve's bison unit to mimic historic fire regimes (Hamilton, 2007). From 2008 to 2010, patches within the Tallgrass Prairie Preserve's bison unit were annually burned and the unit was moderately stocked across a 9400‐ha unit (McMillan et al., 2021).

**TABLE 1:** The total area (ha), elevation (m), typical topography, dominant plant community, range in daily average temperature (°C), and average annual rainfall (cm) between the Joseph H. Williams Tallgrass Prairie Preserve and Wichita Mountains Wildlife Refuge.

Site	Total Area (ha)	Elevation min‐max (m)	Topography	Dominant Plant Community	Daily Average Temperature min‐max (°C)	Average Annual Rainfall (cm)
Joseph H. Williams Tallgrass Prairie Preserve	9400	244–335	Rolling Hills	Tallgrass Prairie	−14.0 – 32.0	95
Wichita Mountains Wildlife Refuge	23,885	422–755	Steep Mountains and Valleys	Mixed Grass Prairie	−12.5 – 36.2	62

*Note*: Daily average temperature and average annual rainfall were obtained from the Foraker and Medicine Park Mesonet stations (https://www.mesonet.org) at the two sites respectively, and represent conditions during the years 2008–2012.

At 23,884‐ha, the Wichita Mountains Wildlife Refuge is made up of several ecosystems that vary with elevation (McMillan et al., 2021), but the grasslands occurring throughout the refuge are characterized as mixed‐grass prairie. Precipitation is much lower on average, but temperatures are similar to the Tallgrass Prairie Preserve (Table 1; Brock et al., 1994; McPherson et al., 2007). The Wichita Mountains Wildlife Refuge is actively managed with prescribed fire and grazing, although unlike the Tallgrass Prairie Preserve, approximately 650 bison and 220 longhorn cattle graze jointly across most of the refuge. From 2010 to 2012, the Wichita Mountains Wildlife Refuge did not have a fixed burn schedule and was lightly stocked with bison and longhorn cattle (McMillan et al., 2021). Although our two study sites differ in topography, precipitation, and plant community structure (Table 1), previous research suggests that bison movement distance through time (i.e., movement rates) may not differ between our two sites (McMillan et al., 2021).

### 2.2 Data collection

For this study, we utilized GPS‐telemetry data collected by The Nature Conservancy and the United States Fish and Wildlife Service from 2008 to 2012 (Allred et al., 2011; McMillan et al., 2021). Staff at the Tallgrass Prairie Preserve collected GPS data from seven females per year from November 2008 to November 2011 (batteries replaced and new individuals chosen November 2009 and 2010), while staff at the Wichita Mountains Wildlife Refuge collected GPS data from six females per year from November 2010 to July 2012 (batteries replaced and new individuals chosen November 2011). Therefore, our dataset included movements from a total of 33 individual plains bison. All plains bison were fitted with either GPS7000MU or GPS3300L model Lotek GPS collars (see Allred et al., 2011). All handling and collar fitting was done by Nature Conservancy or United States Fish and Wildlife employees, and was overseen by large‐animal veterinarians in line with each organization's typical handling procedures. Collar location data were recorded at intervals ranging from 1 h to 2 min during our study period (Allred et al., 2011). The median GPS sample rate was 12 min across all of our data, therefore, we analyzed all movement data at that temporal resolution. Further, our 12 min sampling rate most closely matched the temporal resolution of weather data collected at our two sites. Our use of fine‐scale movement data also reduced the likelihood that our observations were biased by fence‐effects. We did not control for cow‐calf effects on movement. GPS data were differentially corrected prior to our analysis (Allred et al., 2011), using data from nearby base stations.

**FIGURE 1:**
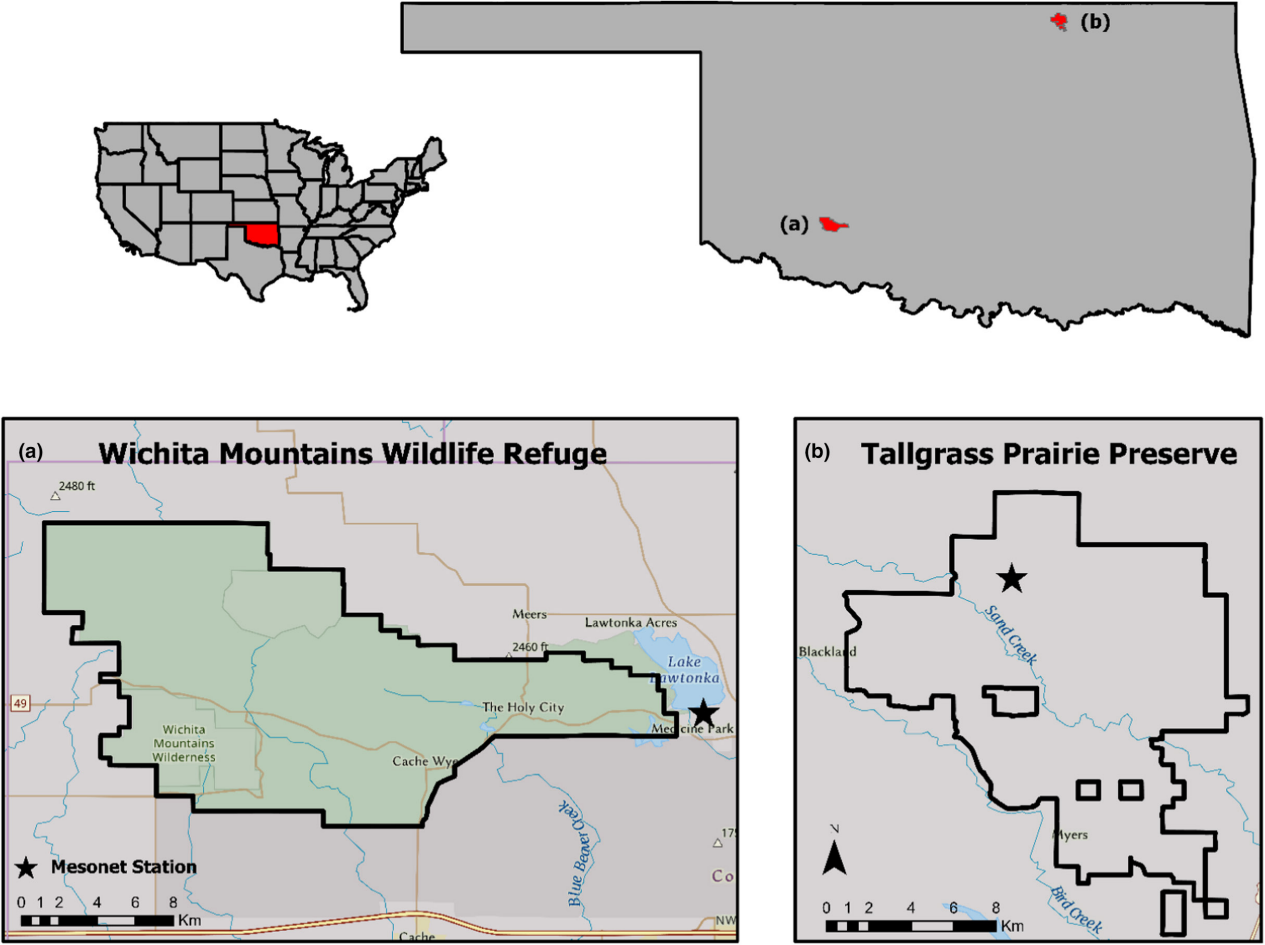
Map showing the position of the two Mesonet Stations that we used to collect weather data relative to our two study sites: (a) the Wichita Mountains Wildlife Refuge and (b) the Joseph H. Williams Tallgrass Prairie Preserve. Also depicted are the relative locations of our two study sites within Oklahoma and the United States of America.

To analyze how bison movements are affected by weather, we paired each 12‐min movement with corresponding weather station data collected at our two sites. We specifically used data from the Foraker Mesonet weather station located within the boundary of the Tallgrass Prairie Preserve (Figure 1), and the Medicine Park Mesonet located approximately 2.8 km from the Wichita Mountains Wildlife Refuge (Figure 1). The Mesonet system was established so that each weather station broadly represents the surrounding area (i.e., soils, elevation, etc.; Brock et al., 1994; McPherson et al., 2007). Therefore, we did not collect or analyze data regarding how bison respond to fine‐scale habitat conditions (e.g., collar‐based temperature data associated with the movement of each individual), but rather how movement changed with broad weather conditions across our sites. We collected 2‐meter air temperature, 10‐meter wind speed, wind direction, relative humidity, solar radiation, 24‐h rainfall accumulation, and daily calibrated soil temperature (5 and 25 cm depth) data from November 2008 to November 2010 and November 2010 to November 2012 for the Foraker and Medicine Park weather stations, respectively. We used calibrated soil temperature data at two depths (5 and 25 cm) to calculate daily fractional water index (Illston et al., 2008) values as a way to estimate broad drought conditions at each site as well.

### 2.3 Data analysis

To investigate potential influences of weather and drought on bison movement patterns, we calculated movement distance from our GPS data, and related movement distance to corresponding weather data collected at each site. We used the package “amt” in R version 4.1.2 (R Core Team, 2021; Signer et al., 2019) to clean and process our GPS‐data prior to analysis. Specifically, we reviewed the dataset to ensure no critical observation information (i.e., latitude, longitude, or timestamp data) was missing and that it did not contain any duplicates. We used the *make_track* function in the package “amt” to create movement tracks from GPS locations for each individual. We then resampled our movement tracks to ensure that each represented an uninterrupted 12‐min sequence of steps (i.e., bursts) using the functions “track_resample” and “steps_by_burst” in the package “amt” and calculated the distance traveled for each 12‐min movement using the function “step_length” (Signer et al., 2019). Spurious movements were removed from our dataset through visual inspection prior to further analysis. With the exception of rainfall and soil temperature data (each reported as daily summaries), all other primary weather metrics were recorded in 5‐min intervals. We paired each movement with the nearest 5‐min weather observation to overcome the timing offset between the movement and weather data used in this study.

**FIGURE 2:**
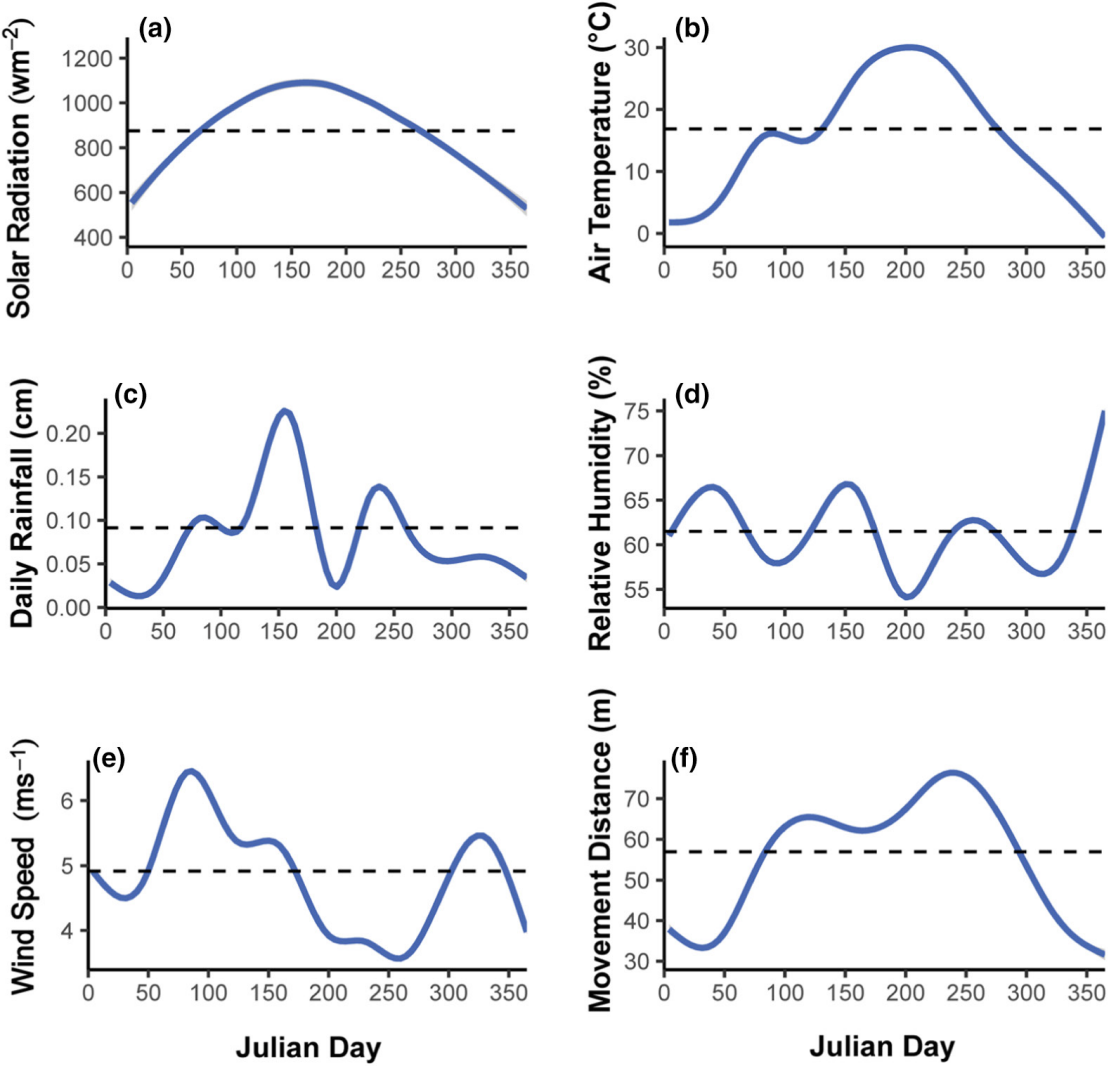
Average (a) max solar radiation (wm^−2^), (b) air temperature (°C), (c) daily rainfall (cm), (d) relative humidity (%), (e) wind speed (ms^−1^), and (f) plains bison movement distance (m) observed for each day of the year (i.e., Julian day) across our two sites. Movement distances represent data collected from 33 female plains bison (*Bison bison* L.) from the Joseph H. Williams Tallgrass Prairie Preserve (November 2008 – November 2011) and the Wichita Mountains Wildlife Refuge (November 2010 – July 2012).

We explored the relationships between plains bison movement, weather, and drought using generalized additive mixed models. Specifically, we fit all reasonable combinations of additive models with multiple weather parameters as fixed effects to analyze the effect of weather on bison movement. We analyzed the effect of drought on plains bison movement by fitting each drought metric individually in single fixed effect models. We fit smoothed predictors (i.e., 2‐m air temperature, 10‐m wind speed, relative humidity, solar radiation, 24‐h rainfall accumulation, and daily fractional water index at 5 and 25 cm soil depth) in our generalized additive mixed models using a cubic spline smoothing basis. Since we treated wind direction as categorical, it was always fit as a parametric (i.e., unsmoothed linear) predictor. Movement distance (i.e., displacement) data tends to be right skewed due to a higher frequency of short movement distances. Thus we fit Gamma distribution models with a log‐link function, which did not require us to perform any data transformations prior to analysis or for interpretation. We confirmed that our data met the assumptions of a Gamma distribution with a log‐link by visually inspecting residual plots (i.e., Q‐Q plot, residuals vs. fitted, residuals vs. linear predictors, etc.) using the package *mgcv*. We accounted for potential variation among individuals, as well as repeated measures for any one individual and across sites, by using the individual ID nested within site as a random intercept in all models. Season and time‐of‐day have a significant influence on plains bison movement across our two sites (McMillan et al., 2021), and are likely to confound any model of weather effects on their movement. Solar radiation is strongly correlated with season (Figure 2) and time of day, and therefore, we accounted for seasonal and diurnal effects on movement by using solar radiation as a random effect in our models. Using solar radiation is likely more biologically informative than using discrete seasonal or day‐night categories, as wildlife movement patterns often violate human‐defined temporal groupings. We transformed our continuous solar radiation data into four discrete groupings to represent the lower (0%–25%), middle (25%–50% and 50%–75%), and upper (75%–100%) quantiles of observed conditions. We then ranked models for each analysis (i.e., weather and drought) using the Akaike information criterion corrected for small samples (AICc). We assessed the fit of our most supported candidate models by visually inspecting residual plots (i.e., residuals vs. fitted values, and residuals vs. predictor variables) ensuring that models did not violate mean–variance or response variable independence assumptions (Wood, 2017). We further assessed model fit by checking the basis dimension (i.e., *k*) and partial residuals of each smoothed parameter (Wood, 2017). Basis dimension values for each smoothed parameter were obtained using the “gam. check” function in the package *mgcv* (Wood & Wood, 2015). Although generalized additive mixed models are useful in analyzing nonlinear data, they often do not provide outputs that are useful for statistical inference. Therefore, we employed breakpoint regression analysis using the package *segmented* (Vito, 2008) to obtain estimated breakpoints and coefficients from individual splines, enabling more detailed statistical inference.

## 3 RESULTS

We analyzed a total of 715,344 12‐min movements from 33 female plains bison, averaging approximately 21,677 12‐min movements per individual, across two sites in Oklahoma. Overall, mean plains bison movement distance across all individuals and years was 56.9 m per 12‐min movement path (SE = 0.1 m) with approximately 28% of the total observed movements exceeding the mean distance (Figure 2). Average daily air temperature across our two study sites ranged from −11.2°C to 34.7°C (Figure 2). Average daily rainfall ranged from 0.00 to 1.57 cm across our two sites, and average windspeed observed per day ranged from 1.61 to 9.69 ms^−1^ (Figure 2). Average daily 25 cm FWI across our two sites ranged from 0.32 to 0.97, and averaged 0.78 throughout our entire study period.

### 3.1 Response to weather

**FIGURE 3:**
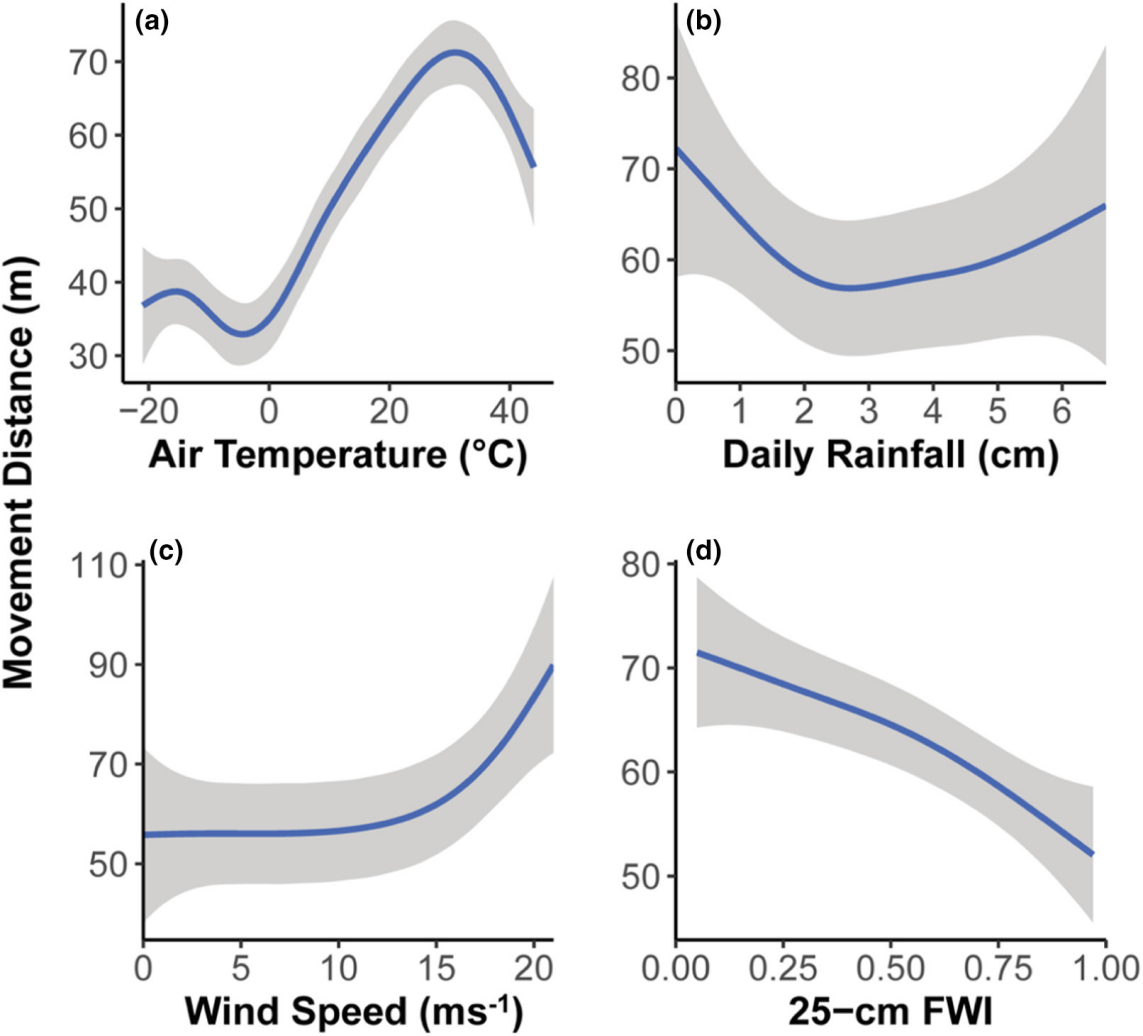
The average distance moved (m) by our female plains bison (*Bison bison* L.) every 12‐min relative to concurrent (a) air temperature (°C), (b) daily rainfall (cm), (c) wind speed (ms^−1^), and (d) 25 cm fractional water index at the Joseph H. Williams Tallgrass Prairie Preserve and the Wichita Mountains Wildlife Refuge from November 2008 to November 2010 and November 2010 to November 2012, respectively. Fractional water index values are correlated with soil moisture, ranging from 0 to 1, representing powdery dry and fully saturated soils respectively. Lines were fit using generalized additive mixed models with a cubic spline smoothing basis, and represent predicted values of each variable while holding all others constant. Shaded area represents a 95% confidence interval around the fitted mean. Each model was fit holding all other parameters at their respective means.

Air temperature better explained plains bison movement distances compared to the other weather parameters we tested in our single fixed‐effect models. Air temperature also had the strongest effect of any single weather parameter we tested (*R* ^2^ = 0.019; Table 2). Movement increased 92.5% with every 10°C increase in air temperature from −21.3°C to 28.2°C (β = 0.023, 95% CI = 0.023, 0.024; Table 3; Figure 3). However, movement decreased 48.5% with every 10°C increase from 28.3°C to 44.3°C (β = −0.012, 95% CI = −0.015, −0.010; Table 3; Figure 3).

**TABLE 2:** Candidate set of generalized additive mixed models explaining the additive effect of air temperature, daily rainfall, wind speed, relative humidity, and wind direction on female plains bison (*Bison bison* L.) movement distance.

Model	∆Log‐Likelihood	K	∆AIC_c_	AIC_c_ Weight	Adj. *R* ^2^	Deviance Explained (%)
Air Temperature + Daily Rainfall + Wind Speed	7216.6	3	0.0	1.0	0.02	3.11
Air Temperature + Daily Rainfall	6421.8	2	1576.2	< 0.1	0.02	3.03
Air Temperature + Wind Speed	5955.7	2	2504.6	< 0.1	0.02	3.00
Air Temperature + Relative Humidity	5731.1	2	2958.2	< 0.1	0.02	3.00
Air Temperature	5169.2	1	4064.4	< 0.1	0.02	2.93
Daily Rainfall + Wind Speed	2090.2	2	10,236.0	< 0.1	0.01	1.50
Wind Speed	1334.6	1	11,730.0	< 0.1	0.01	1.41
Wind Direction	1193.9	1	12,003.3	< 0.1	0.01	1.37
Daily Rainfall	1189.6	1	12,022.7	< 0.1	0.01	1.44
Relative Humidity + Wind Speed	755.5	2	12,905.9	< 0.1	0.01	1.51
Null	430.7	0	13,523.7	< 0.1	0.01	1.35
Rainfall + Relative Humidity	264.2	2	13,891.4	< 0.1	0.01	1.56
Relative Humidity	0.0	1	14,402.8	< 0.1	0.01	1.45

*Note*: Movement data were collected every 12‐min from November 2008 to November 2010 and November 2010 to November 2012 at the Joseph H. Williams Tallgrass Prairie Preserve and the Wichita Mountains Wildlife Refuge, respectively. Models were fit with individual ID and site as random effects to account for variability present among individuals, as well as repeated movement measures for each individual. Solar radiation was also fit as a random effect in all models to account for seasonal and diurnal effects on movement. Model parameters were fit with a cubic spline smoothing basis, except wind direction which was always fit as a linear predictor (i.e., was not smoothed).

**TABLE 3:** Summary of fixed effects from our most supported generalized additive mixed models predicting the effects of weather and drought on fine‐scale (12‐min) mean movement distance of female plains bison (*Bison bison* L.) at the Joseph H. Williams Tallgrass Prairie Preserve and Wichita Mountains Wildlife Refuge.

Air Temperature + Daily Rainfall + Wind Speed				
	β	95% CI	Back‐Transformed Estimate	Change (%)
Air Temperature (°C)				
−21.30–28.20	0.023	(0.023, 0.024)	9.25 m per 10.0°C	92.50
28.30–44.30	−0.012	(−0.015, −0.010)	−4.85 m per 10.0°C	−48.50
Daily Rainfall (cm)				
0.00–0.18	1.305	(1.090, 1.521)	14.97 m per 0.1 cm	149.70
0.19–6.68	−0.020	(−0.034, −0.004)	−0.11 m per 0.1 cm	1.10
Wind Speed (ms^−1^)				
0.00–6.60	−0.006	(−0.009, −0.003)	−0.35 m per 1.0 ms^−1^	−0.35
6.61–21.00	0.012	(0.008, 0.017)	0.72 m per 1.0 ms^−1^	0.72
25 cm FWI				
Drought				
0.00–1.00	−0.363	(−0.379, −0.347)	−2.32 m per 0.1 FWI	−23.20

*Note*: Coefficients were obtained using breakpoint regression, and are meant to facilitate statistical inference from our models. Note: percent (%) change represents the change in movement distance (m) per every 1‐unit increase for each parameter measured.

Our most supported model for describing the relationship between plains bison movement and weather parameters included the additive effect of air temperature, rainfall, and wind speed as smoothed fixed‐effects (*R* ^2^ = 0.02; Table 2). We further validated our most supported model by ensuring it significantly improved model fit compared to the null (likelihood ratio test, *p* < .01). Movement distance increased 149.7% with every 0.1 cm increase in daily rainfall from 0.00 to 0.18 cm per day (β = 1.305, 95% CI = 1.090, 1.521), but decreased 1.1% with every 0.1 cm increase in daily rainfall from 0.19 to 6.68 cm per day (β = −0.019, 95% CI = −0.034, −0.004; Table 3; Figure 3). Movement distance decreased 0.35% with every 1 ms^−1^ increase in wind speed from 0.00 to 6.60 ms^−1^ (β = −0.006, 95% CI = −0.009, −0.003), but increased 0.72% with every 1 ms^−1^ increase from 6.70 to 21.00 ms^−1^ (β = 0.012, 95% CI = 0.008, 0.017; Table 3; Figure 3).

### 3.2 Response to drought

Our most supported model suggests that variability in plains bison movement was better explained by changes in 25 cm fractional water index (i.e., an index of soil moisture conditions; FWI) compared to measurements shallower in the soil profile (i.e., 5 cm FWI; Table 4). We found that plains bison movement distance was highest (est. = 74.96 m) when soils were powdery dry (i.e., FWI = 0.00), and decreased 23.2% with every 0.10 increase in FWI (Table 3; Figure 3).

**TABLE 4:** Candidate set of generalized additive mixed models explaining the effect of drought (i.e., using fractional water index, or FWI) on female plains bison (*Bison bison* L.) movement distance.

Model	∆AIC_c_	AIC_c_ Weight	Adj. *R* ^2^	Deviance Explained
25‐cm FWI	0.0	1.0	0.014	2.05
5‐cm FWI	2441.1	<0.001	0.013	2.06
25‐cm FWI*	3112.0	<0.001	0.012	1.71
5‐cm FWI*	3922.7	<0.001	0.012	1.85
Null	3942.9	<0.001	0.009	1.35

*Note*: Movement data were collected every 12‐min from November 2008 to November 2010 and November 2010 to November 2012 at the Joseph H. Williams Tallgrass Prairie Preserve and the Wichita Mountains Wildlife Refuge, respectively. FWI was calculated using daily soil moisture data from the two sites. Models were fit with individual ID as a random effect to account for variability present among individuals, as well as repeated movement measures for each individual. Model parameters marked with an asterisk were fit as linear predictors. Otherwise, model parameters were fit with a cubic spline smoothing basis.

## 4 DISCUSSION

Our study supports the hypothesis that weather influences fine‐scale American plains bison movements. Air temperature had the strongest effect on 12‐min plains bison movement distance compared to any other single weather parameter we tested (Table 2). Plains bison movements were the shortest at extremely low temperatures (i.e., <−20°C), perhaps due to physiological demands of movement during those times (Sheppard et al., 2021). However, plains bison moved further with increasing air temperature (i.e., −21°C to 28°C), suggesting that they may be tracking thermally dependent, fine‐scale, changes in photosynthesis (Pilarski, 1999; Sage & Kubien, 2007)—that is, forage quality—as well as favorable physiological conditions. We also found that during times of excessive heat (i.e., 28°C to 44°C), plains bison movements declined, suggesting a physiological threshold on movement. Excessive heat has been shown to restrict plains bison movement on the landscape as they seek thermal refugia—often riparian areas—to escape extreme heat (i.e., >39°C; Allred et al., 2013). Our plains bison response was also similar—both in direction and effect size—to the reported effects of air temperature on wood bison in Canada (Sheppard et al., 2021), potentially suggesting a similar response, regardless of subspecies, across North America. Air temperature has strong direct (via physiological effects, through increased energetic and nutrient demands; Martin & Barboza, 2020) and indirect (e.g., temperature‐driven changes in forage quality; Sage & Kubien, 2007) effects on where and how other ungulates move across landscapes as well (Schmidt et al., 2016; van Beest et al., 2012; van Beest et al., 2013). Our results add to a growing body of evidence supporting that weather not only directly affects where animals move, but also how they move across landscapes (Rivrud et al., 2010; Schmidt et al., 2016; van Beest et al., 2011; van Beest et al., 2013).

The additive effect of air temperature, wind speed, and daily rainfall best predicted plains bison movement distance compared to the other combinations of weather parameters we tested. Although wind speed and daily rainfall were included in our most supported model, their effect was minimal (e.g., decreased 0.35% with every 1 ms^−1^ increase in wind speed from 0.00 to 6.60 ms^−1^; and increased 0.72% with every 1 ms^−1^ increase from 6.70 to 21.00 ms^−1^), and highly variable (Figure 3). However, wind speed and daily rainfall may have a stronger influence on other movement parameters not measured (e.g., sinuosity). Wind speed and rainfall can influence spatial and temporal patterns of forage quantity and quality across landscapes (e.g., species composition and structural differences on exposed versus sheltered landscapes), therefore influencing behavioral patterns and resource selection. Wind speed in particular can influence behavioral patterns of some ungulates, with red deer (*Cervus elaphus* L.) switching from foraging to sheltering behaviors depending on wind speed in the winter months (Conradt et al., 2000). Red deer response was also shown to differ with sex, where males were more sensitive to low temperatures and wind than females (Conradt et al., 2000). Since we did not collect movement data from male plains bison, our data are limited to female response to weather. Although plains bison exhibit sex‐specific behaviors (e.g., sexually segregated herd structure throughout much of the year; Lott, 2002), it is unknown if their response to weather changes with sex. Future studies of plains bison should further investigate how other primary and secondary movement parameters, and behavior, are affected by weather across individual demographics (i.e., sex, age).

In line with our predictions, we found that drought conditions deeper in the soil profile (i.e., severe drought) better predicted plains bison movements compared to those at shallower depths (i.e., less severe drought). Historical accounts of movement patterns in plains bison suggest they may have traveled long distances, and for multiple days without water (Hornaday, 1889). More recent research has also suggested that plains bison may not be very sensitive to drought or surface water distribution across the landscape relative to other domestic ungulate grazers (Kohl et al., 2013). Our results support that plains bison are likely tolerant to short‐term drought conditions, as evident by shallower moisture conditions in the soil profile. However, that 25‐cm FWI better predicted distance moved than both shallower soil moisture (5‐cm) and the null model suggests that they are not immune to the effects of more extreme drought. Although drought can influence forage quantity and quality, forages can also provide ungulate grazers with some (or nearly all) of their daily water requirement (Kay, 1997; King, 1983). Plant moisture content is contingent upon soil moisture, and during severe drought, ungulate grazers must obtain their water requirements from permanent or ephemeral water sources (Kay, 1997). As drought becomes more intense, plant growth and photosynthesis rates decline (Chaves et al., 2003), and high‐quality forage becomes spatially limited through time. Therefore, plains bison experiencing severe drought conditions likely move greater distances in search of areas to balance their energetic (nutrient and water) requirements.

Although our study represents the first multiherd assessment of plains bison movement response to weather, the patterns we observed may not be absolutely replicated outside of the southern Great Plains. Specifically, the thermal extremes we observed are quite different from those likely to occur in the central or northern Great Plains, and plains bison there may exhibit different behaviors based on acclimation to those extremes. However, the general pattern (and strength) of response that we observed from plains bison in Oklahoma was similar to those observed in wood bison (*Bison athabascae* Rhoads) in Canada (Sheppard et al., 2021). It is possible that our collective datasets reflect a general physiological response across the *Bison* genus, but further work will be needed to verify that hypothesis. Weather and drought, overall, only weakly explained the overall variation in bison movement that we observed—that is, the deviance explained from the most supported models was 3.11 and 2.05% for weather and drought, respectively. Therefore, it is also possible that other interactive effects between weather and the physical landscape better explain our observed plains bison movements than weather alone—especially across large heterogeneous landscapes.

When confronted with ambient physiological stress, plains bison are faced with two choices to mitigate that stress: (1) move to a new place on the landscape where the stress is relieved or avoided (Allred et al., 2013) or (2) acclimate to the current condition. Prior to development and westward expansion, when extreme drought or inhospitable weather patterns occurred across expansive landscapes, plains bison would have been able to freely move great distances in search of more hospitable conditions. However, plains bison are now relegated to relatively small, homogenously managed, fenced landscapes that are often privately owned. As we move through the Anthropocene, changes in climate are predicted to accelerate beyond the ability of many species to adapt, resulting in range shifts (Cahill et al., 2013; Thomas, 2010) and local species extinctions (Duncan et al., 2012). However, for large ungulates like plains bison that are adapted to a wide range of ecosystems, the threat may be more related to restrictions to movement (e.g., fragmentation, urbanization), as long distance movements to avoid or moderate weather extremes are no longer an option. Even in vast landscapes like Yellowstone National Park (899,116 ha) where a considerable portion of the nearly 5000 resident plains bison annually disperse from the park, such movements are restricted or discouraged through culling or hazing (Plumb et al., 2009). Similar conflicts surrounding movement exist for other ungulates across the globe, including elk in North America (Frair et al., 2005) and elephants in Africa (*Loxodonta africana* Blumenbach) and Asia (*Elephas maximus* L.; Shaffer et al., 2019). This further highlights the complexity of developing conservation efforts to mitigate climate change impacts on large ungulates. Our results suggest that facilitating increased movement may be key to sustaining plains bison and other large ungulates in the future, even across vast landscapes (e.g., Yellowstone National Park or Kruger National Park), as they will likely move greater distances (potentially beyond park boundaries) as temperatures warm, and droughts become more frequent, severe, and longer lasting. Many of the world's existing large conservation areas are arranged, or managed, in ways that harbor very little ecological resiliency under a changing climate (Fuhlendorf et al., 2018; Holling & Meffe, 1996). Moreover, as new ambitious rewilding and restoration efforts are proposed and implemented (Fuhlendorf et al., 2018), few include actions based around increasing ecological resiliency (Holling & Meffe, 1996) through restoring ecological processes (e.g., fire) as well as keystone species. In the case of keystone species such as plains bison and other large ungulates, the impact of weather, especially under a changing climate, may significantly limit grassland restoration efforts. In particular, weather‐driven alterations in ungulate movement have the potential to affect grassland structure and function via changes to disturbance frequency, timing, and intensity. Changes to grassland herbivory‐vegetation feedbacks, for example, can have cascading effects relevant to ecosystem function and conservation (e.g., increased fire threat, woody plant encroachment; Fuhlendorf & Engle, 2001; Werner et al., 2020). Therefore, understanding how large ungulates respond to climate change will critically inform many biodiversity conservation efforts throughout the Anthropocene.

## 5 CONCLUSIONS

American plains bison movement distances that we observed were better explained by the additive effect of air temperature, wind speed, and daily rainfall compared to other weather parameters. Movement distances were also better explained by severe drought (i.e., drought conditions deeper in the soil profile) than moderate drought conditions. Although weather and drought alone did not explain much of the total variance of our movement data (3.11% and 2.05% deviance explained, respectively), our study adds to a growing line of evidence that weather should be considered in future assessments of plains bison movement. Animal movement is affected by complex interactions between physical (e.g., topography, water distribution, patterns of forage) and ambient (e.g., thermal) landscape variables, as well as individual physiological conditions. Understanding how these complex interactions influence movement will be critical to the conservation of many large, and important, species; especially as efforts are complicated by urbanization, landscape fragmentation, and climate change.

## AUTHOR CONTRIBUTIONS

**Nicholas A. McMillan:** Conceptualization (equal); data curation (equal); formal analysis (lead); investigation (lead); methodology (equal); project administration (equal); visualization (lead); writing – original draft (lead); writing – review and editing (lead). **Samuel D. Fuhlendorf:** Conceptualization (equal); data curation (equal); funding acquisition (lead); investigation (equal); methodology (equal); project administration (equal); supervision (lead); visualization (equal); writing – original draft (supporting); writing – review and editing (supporting). **Barney Luttbeg:** Conceptualization (supporting); formal analysis (supporting); methodology (equal); writing – original draft (supporting); writing – review and editing (supporting). **Laura Goodman:** Conceptualization (supporting); investigation (supporting); methodology (supporting); writing – original draft (supporting). **Craig Davis:** Conceptualization (supporting); formal analysis (supporting); investigation (supporting); visualization (supporting); writing – original draft (supporting); writing – review and editing (supporting). **Brady Allred:** Conceptualization (equal); data curation (lead); writing – original draft (supporting). **Robert G. Hamilton:** Conceptualization (equal); funding acquisition (lead); investigation (equal); project administration (lead); resources (lead).

## CONFLICT OF INTEREST

The authors have declared no conflict of interest.

## Data Availability

The movement and weather data that support these findings are archived and publicly available in Dryad (https://doi.org/10.5061/dryad.547d7wmcq).
